# The Relationship Between Educational Years and Phonemic Verbal Fluency (PVF) and Semantic Verbal Fluency (SVF) Tasks in Spanish Patients Diagnosed With Schizophrenia, Bipolar Disorder, and Psychotic Bipolar Disorder

**DOI:** 10.1097/MD.0000000000001596

**Published:** 2015-10-02

**Authors:** Eduardo García-Laredo, Fernando Maestú, Miguel Ángel Castellanos, Juan D. Molina, Elisa Peréz-Moreno

**Affiliations:** From the Faculty of Psychology (EG-L), Basic Psychology II Department, University Complutense; Laboratory of Cognitive and Computational Neuroscience (FM), Centre of Biomedical Technology (CTB), Complutense University of Madrid (UCM) and Technological University of Madrid (UPM); Faculty of Psychology (MAC, EP-M), Department of Methodology of Behavioral Sciences, University Complutense; and Acute Inpatients Unit (JDM), Dr R. Lafora Psychiatric Hospital, Madrid, Spain.

## Abstract

Semantic and verbal fluency tasks are widely used as a measure of frontal capacities. It has been well described in literature that patients affected by schizophrenic and bipolar disorders present a worse execution in these tasks. Some authors have also noted the importance of educational years. Our objective is to analyze whether the effect of cognitive malfunction caused by apathology is superior to the expected effect of years of education in phonemic verbal fluency (PVF) and semantic verbal fluency (SVF) task execution.

A total of 62 individuals took part in this study, out of which 23 were patients with schizophrenic paranoid disorder, 11 suffered from bipolar disorder with psychotic symptomatology, 13 suffered from bipolar disorder without psychotic symptomatology, and 15 participants were nonpathological individuals. All participants were evaluated with the PVF and SVF tests (animals and tools). The performance/execution results were analyzed with a mixed-model ANCOVA, with educational years as a covariable.

The effect of education seems to be more determined by PVF FAS tests than by SVF. With PVF FAS tasks, the expected effect of pathology disappears when the covariable EDUCATION is introduced. With SVF tasks, the effect continues to be significant, even though the EDUACTION covariable dims such effect.

These results suggest that SVF tests (animals category) are better evaluation tools as they are less dependent on the patients’ education than PVF FAS tests.

## INTRODUCTION

Verbal fluency tasks (VF tasks) were initially used as an objective measurement of the linguistic capacity of the individual.^1^ Afterward they were popularized as evaluation tools for patients with cerebral damage.^2^ More recently, they have been specially used as an evaluation of the functioning of the frontal lobe's cognitive capacities.^3^

Despite its apparent simplicity, the use of these tests as a measurement of frontal capacities is due to the fact that its execution entails, beyond the intervention of the linguistic processing and the capacity to start and maintain the production of words, other cognitive components such as attention, short-term memory, cognitive flexibility, response inhibition capacity, processing speed, and semantic memory.^4–6^ Owing to the simplicity of its application, they have been frequently used in the evaluation of executive functioning, especially in the study and diagnosis of cognitive deterioration in dementia and *traumatic brain injury*. They have also been used in other pathologies in which neurocognitive processes are affected, such as schizophrenia, bipolar disorder, or depression.^7–9^

Although VF is a frontal function, some authors^10^ doubt to what extent the VF could be considered as an executive function. Nevertheless, it is widely accepted that VF tasks allow objectifying the capacity of the individual to generate internal strategies—the aim is to reach a goal. These tasks are widely used in neuropsychological evaluation as a measurement of executive dysfunction.

### VF Test Applications

The application of these tests consists of asking the evaluated individuals to say out loud as many words as they can. They must say them according to a specific criterion, under a time restriction—usually to 60 seconds.^11^ The most commonly used tests are as follows.

#### Phonemic verbal fluency (PVF)

The evaluated individual must produce words that begin with certain letters, frequently F-A-S (PVF FAS). They cannot use either derivatives of the same word or first names. Although the letters F-A-S are also used among Spanish speakers, there are some authors,^12,13^ who have proposed the use of other letters (P, M, and R) in order to minimize the effects of language. Nevertheless, the use of these letters is less common.

#### Semantic verbal fluency (SVF)

It is another very common application. It implies production of words, regardless of the initial letter. These words must belong to the same category—the “Animals” category is more frequently used. In addition, studies on this category have indicated that its use is suitable for the assessment of Spanish speakers, regardless of their country of origin and the influence of cultural differences.^14^

Despite the fact that both applications share common elements, some authors suggest that they imply different processes. The PVF task would be an uncommon task for the individual, who would require a major cognitive effort to inhibit incorrect answers.^15^ However, in SVF tasks, the evocation of words belonging to a specific category is centered on the meaning of the words, therefore depending more on semantic memory.^16^ In this respect, Gourovitch et al^17^—by means of neuroimage technologies (18 normal controls were studied with oxygen-15 water regional cerebral blood flow positron emission tomography)—found major activity in the prefrontal lobe in PVF tasks and major activation of the temporary cortex in SVF tasks.

Although some authors, such as Rende, Ramsberger, and Miyake,^18^ have noted differences between both tasks of VF in short-term memory, they have also highlighted executive function as a common process between them. The relevance of executive function is confirmed by studies with patients suffering from a frontal lobe injury.^19,20,16^ These patients present comparable alterations for both tasks.

Even though both tasks involve executive functioning, the differences among the other processes involved can have consequences in the diagnosis of dementia. For example, Monsch et al^21^ believed that SVF is a better discriminator for schizophrenia than PVF. This discriminative superiority is due to the test's high dependence on the structures of semantic knowledge, which can be deteriorated in the first phases of Alzheimer dementia. In this sense, there are authors who support that SVF allows to classify—with sufficient accuracy—individuals with and without dementia.^22^

Other forms of application of VF tasks exist, such as the excluded letter fluency tasks in which words that do not contain certain indicated letters are evoked.^23^ Some authors have suggested the use of A, E, and S as excluded letters for Spanish speakers.^13^ Another application would be the fluency of actions tasks, where the aim is to evoke the greatest possible number of words that designate an action,^24^ or the fluency of peoples’ names, which is presented as a test less affected by socio-educational variables.^25^ Although the study of these tests is interesting, their use is less frequent than the use of phonemic and semantic tasks.

### VF and Years of Education

Despite its simplicity and its wide use, research among the Spanish-speaking population suggests that variables—such as sex, age, and, essentially, educational years—could have a significant weight on its performance/execution.^26^ In this sense, Benito-Cuadrado et al^27^ noted that the animal semantic test on Spanish population presented a significantly high correlation with years of formal education. Casals-Coll et al,^13^ on Spanish population studies, corroborate the effect of education on VF tasks. Moreover, it has also been shown that age has a scarce effect and that sex has a minimum effect on the performance of these tasks (179 cognitive stable participants, between 18 and 49 years of age). Lozano and Ostrosky-Solís,^28^ on Mexican population studies, have found that schooling level explained a major percentage of the variance in comparison to the age. No significant effect has been found for sex (2221 participants, functionally independent, aged from 6 to 96 years). These results coincide with those of Ostrosky-Solis's study^14^ where no differences of execution between Spanish-speaking samples in different cultural areas were found. However, differences were found for years of education, being especially relevant in young subjects with a high educational level.

### Limits of the VF Task

Frequently, literature reviews regarding the effect of educational years on the execution in VF tests by Spanish speakers have generally presented normative data from nonpathological populations, with no previous evidence of possible executive dysfunctions. Because there is a high generalized use of these tests for the evaluation of patients with pathologies—that have alterations in frontal executive functioning—the study gains a different importance and deepens the knowledge on whether the effect of executive dysfunction is superior to the effect that it could be expected from educational years in individuals with no pathology—both on the FVI FAS test and on FVS test. With this aim, the effect of the FVI FAS and FVS—animals and tools category—was studied. This was performed with control individuals and individuals suffering from psychiatric pathologies frequently associated to executive dysfunction: patients affected by a schizophrenic disorder, or bipolar disorder—with and without psychotic symptomatology.

### Education and VF. Lexical/Semantic Store or Cognitive Reserve?

A possible way to observe the effect of education in VF tasks may be through the development of the lexical/semantic memory store. A better education would imply greater and more sophisticated lexical/semantic stores, which would yield better performances in these tasks. A second way to check for effects would be related to the concept of cognitive reserve, where both structural cerebral factors and functional factors (intellectual activity, years of education, profession, or general intelligence) would lead the individual to possess a greater efficiency in his neural processes and to have a greater aptitude to develop alternative cerebral networks that could—to certain degree—protect, and even compensate the effects of aging or cerebral pathologies.^29,30^

The effect of cognitive reserve has been scarcely investigated in bipolar disorder, with occasionally contradictory results. This may not be surprising because there are authors who doubt whether cognitive alterations precede the disease or are a consequence of it.^31^ They also doubt whether cognition during symptom remission remains relatively intact,^32^ or whether it will continue presenting lasting cognitive dysfunctions. In this sense, Martínez-Arán et al^33^ noted that this happened in approximately a third of patients, presenting lasting cognitive dysfunctions.

The effect of cognitive reserve in schizophrenia has been slightly more studied than in the bipolar disorder. Reichenberg et al,^34^ in a cohort study, using intellectual measurements, language, and behavioral functioning data from individuals aged between 16 and 17 years obtained from the Israeli Draft Board Registry and the Israeli National Psychiatric Hospitalization Case Registry, found that the individual suffering from schizophrenia showed significant premorbid deficits in intellectual measures. These deficits were also found for behavioral functioning and writing comprehension. These performances were significantly worse than those obtained by individuals with nonpsychotic bipolar disorder—there were no differences between them and control individuals. Other authors also found information that supports the effect of the cognitive reserve in schizophrenia. Patients with a high premorbid IQ in their childhood have a better social and functional long-term evolution,^35^ though this could possibly be tied to a better conscience of the disease^36^ and a better use of health resources.^37^

However, the study of the effect of cognitive reserve in schizophrenia is a complex task because the neurodevelopment attached to the disease would imply that pathological processes would affect the possible cognitive reserve that patients could have gathered. It is difficult to refer to a clear discontinuity between normal and pathological—as may occur with acute cerebral damage cases. In addition, there are authors who consider that cognitive reserve and pathology should not be seen as independent factors.^30^

In addition, another difficulty must be taken into account—when educational years are taken as a referent, it must be considered that, on one hand, it is usual to observe an early appearance of a cognitive deterioration in most patients. This would interfere with their educational successes. On the other hand, the posterior appearance of clinical symptoms leads to the withdrawal of any educational project the patient may have.

Regarding the relation between VF and cognitive reserve, Rami et al^38^ in an experimental study showed that PVF and SVF test—animals category—correlated significantly with elderly individuals who were healthy but affected by Alzheimer disease. This study used a cognitive reserve questionnaire composed of different bibliographic aspects—such as important variables of intellectual activity on the formation of cognitive reserve.

## MATERIALS AND METHODOLOGY

A total of 62 participants were recruited for this study (32 men and 30 women) and distributed into 4 groups: patients suffering from schizophrenic disorder of the paranoid subtype (n = 23, with an average age of 39.56 years and an average course of disease of 15.43 years); patients suffering from bipolar disorder with psychotic symptomatology (n = 11, with an average age of 45.27 years and an average course of disease of 12.5 years); patients suffering from bipolar disorder without psychotic symptomatology (n = 13, with an average age of 44.61 years and an average course of disease of 15 years), and finally, a control group of nonpathological individuals (n = 15, with an average age of 36 years). Patients were attending the brief hospitalization units of the following hospital centers: Hospital D. Rafael Lafora (Madrid), Fundación Hospital de Alcorcón (Alcorcón, Madrid), Hospital Gregorio Marañón (Madrid), and Complejo Asistencial Benito Menni (Ciempozuelos, Madrid). This study was approved by the board of each hospital center. This study involved the patient's written consent.

All patients were stabilized, in remission from their symptoms and met the DSM-IV-Tr^39^ diagnostic criteria for their respective diseases and did not present any comorbid pathologies. The consultation guide of the DSM-5 diagnostic criteria^40^ does not present any new diagnostic criteria for schizophrenic and bipolar disorders that supposed the exclusion of any patient in the experimental group.

The control group was constituted by voluntary individuals not suffering from any psychiatric pathology or drug consumption habits.

All individuals (in all groups) who presented organic deficits that sharply interfered in the suitable accomplishment of the test (organic problems of speech or expression or severe hearing problems) were excluded from the study. The readers of the index tests and reference standard were blind (masked) to the results of the test.

The descriptive characteristics of the years of formal education coursed by the sample are shown in Table 1.

**TABLE 1:**
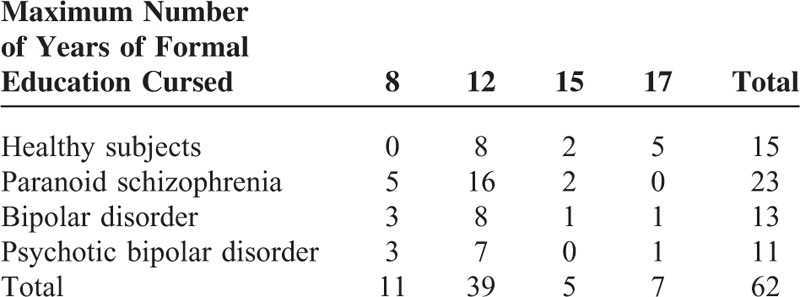
Education Level

## MEASUREMENTS

The participants were evaluated in 2 VF tests, a phonemic one and a semantic one.

*PVF-FAS*: Participants were requested to produce the maximum possible number of words beginning with a specific letter (F, A, and S).
*SVF*: Participants were requested to produce the maximum possible number of words that belong to the “animal” (high fluency) and “tools” (low fluency) categories.

In every test administration, the participants were indicated to follow certain rules: they should not use first names, numbers, derivatives from the same word, or declinations of the same verb. In addition, the time limit for every task was 60 seconds. In the “animals” category, the names of extinguished, imaginary, or magic animals (dinosaur, unicorn, etc.) were permitted, but animal first names (such as King Kong) were not accepted.

## RESULTS

As a measure of phonemic execution, the total number of words produced by the participant in all 3 tasks (F-A-S) was used. As a measure of semantic performance, the total number of words produced in the “Animal” and “Tools” tasks was used. The results of the participants’ execution are shown in Table 2, including their descriptive statistics. In the contrasts results, the partial effect size is represented by and the power by symbol.

**TABLE 2:**
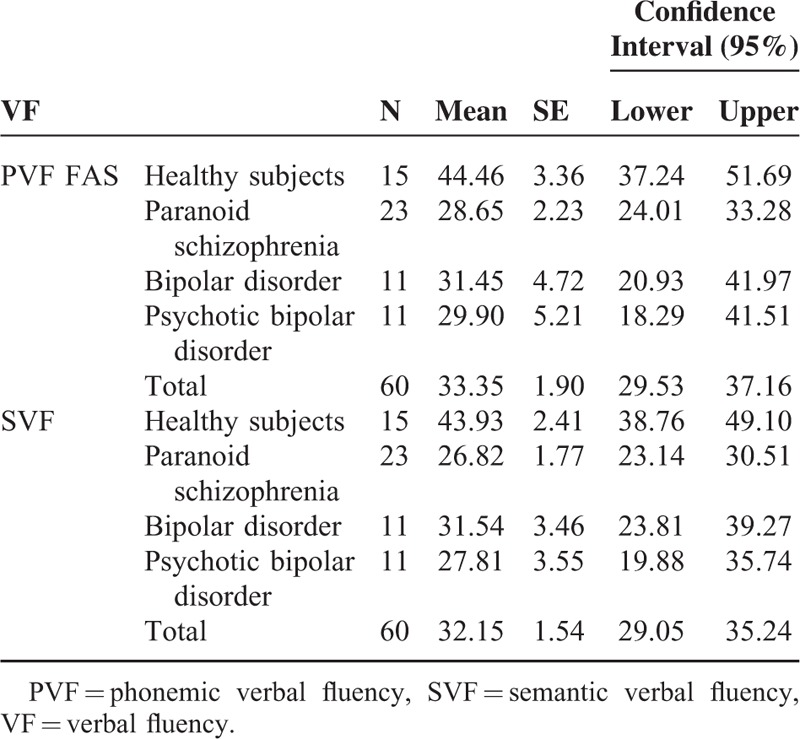
Descriptives VF

Two analyses of variance were calculated for independent groups with the results of every measure. The reasonable fulfillment of the assumptions was verified. For the PVF variable, significant differences between groups, F(3, 56) = 4.566; *P* < 0.006; η^2^ = 0.197; 1-β2 = 0.864, were found. Bonferroni comparisons only showed significant differences between the control group and the schizophrenia patients group. For the SVF variable, differences between groups were also found: F(3, 56) = 9.933; *P* < 0.000; η^2^ = 0.347; 1-β2 = 0.997. Bonferroni comparisons showed differences between the control group and the remaining groups, but there were no differences between patient groups.

To test the effect of education, 2 analysis of covariance were carried out, 1 for each measure. The used software was the package *ez* of the software R.^41^ The variable education was used as a covariable in both cases. The homogeneity of the slopes was tested across the contrasts of the interaction of every measure by the education variable and were found to be nonsignificant in both cases: F(3, 52) = 1.52, *P* < 0.218 and F(3, 52) = 1.24, *P* < 0.30, respectively. The ANCOVA did not show differences either for the FV variable F(1, 56) = 0.682; *P* < 0.411; η^2∗^ = 0.012; 1-β = 0.05, or for the pathology groups F(3, 56) = 2.69; *P* *<* 0.054; η^2∗^ = 0.14; 1-β = 0.52, or for the interaction between pathology groups and FV F(3, 56) = 0.14; *P* < 0.929; η^2∗^ = 0.007; 1-β = 0.07.

Independent analyses for each of the VF measurements did not show the effect of the groups variables concerning the punctuation FVI FAS when education was introduced as a variable F(3, 55) = 1.613; *P* < 0.197; η^2^ = 0.081; 1-β = 0.401, but it was significant for the SVF measure F(3, 55) = 5.099; *P* < 0.003; η^2^ = 0.218; 1-β = 0.902. In this last case, Bonferroni comparisons showed 2 groups: one formed by the control individuals with the highest punctuations, and another formed by individuals with schizophrenia and by bipolar individuals with psychosis symptoms with the lowest punctuations. In an intermediate position, bipolar individuals that showed no differences with other groups were found.

Pearson correlations between formal education years and the punctuations obtained in VF for every group can be seen in Table 3. For the PVF measure, the highest significant correlations were for the group of schizophrenia patients and the bipolar group without psychosis symptoms. For the SVF measure, the only significant correlation was met in the group with schizophrenia.

**TABLE 3:**
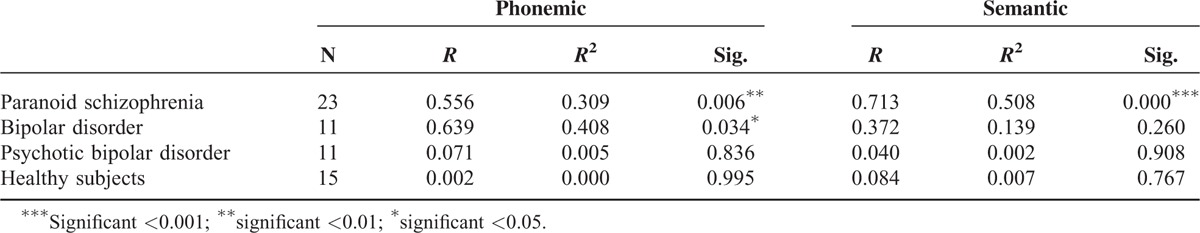
Correlations Between Formal Education Years and Punctuations in Verbal Fluency

## DISCUSSION

In this study, it was evaluated whether education years and age are factors that play a role in the VF performance of a population of patients with schizophrenia and bipolar disorder. In general, the results of this study are similar to those obtained in previous research on the degree of cognitive impairment across psychiatric disorders. Thus, schizophrenic patients showed a worse performance in comparison to the bipolar disorder patients. However, when the bipolar disorder group was divided in those with or without psychotic symptoms, those with these symptoms did not show any difference in their performance in comparison to schizophrenic patients, indicating the relevance of psychotic symptoms. Finally, when years of education were included as a variable for the final analysis, it was clear that the higher the years of education, the better the performance on the phonetic fluency test.

Regarding the role of psychotic symptoms in the performance of the VF test, it is noted that standard errors are notably higher in bipolar patients, in agreement with previous literature. This lack of a uniform pattern could be caused by a limited control of the clinical and pharmacological variables, which constitute too wide a criterium of reference^33^ and the possible existence of residual symptoms of the disease in the *euthymic period*.^42^ It is not rare to associate the psychopathic symptoms with a worse execution of the VF tasks. There exists a generalized neurocognitive deterioration before psychosis appears and the disease is diagnosed, even among 7- to 13-year-old patients.^34^ These deficits will be maintained until they become significantly worse in their transition to psychosis.^43^ Among other domains, this will be associated to a bigger deficit in VF.^44^ These data suggest that these dysfunctions are significant and central to the psychotic disorders,^45^ and without excluding the role of environmental variables after birth, this would go in favor of the neurodevelopmental hypothesis.

A second major finding of this study was the effect of years of education on the performance of the VF test. The lack of differences in performance between the PVF and SVF is not in agreement with previous findings.^7^ In these previous studies, it is usual to find an advantage in phonemic tasks, especially in schizophrenic population. It was observed that these differences were clearer when chronic patients were taken into account. This is an important difference between previous work and the present study as chronic patients were intentionally excluded from our study. Furthermore, Buriel et al^26^ noted that the use of the “tools” category is not an objective and consistent category due to its sociocultural and professional bias.

Previous results seem to show a strong dependence of years of education on both versions of the VF test. In the present study, the effect of education seems to be more determinant on PVF tasks than on SVF tasks, which was also found in other studies.^26^ In PVF, the effect of pathology disappears by introducing education as covariable, whereas in SVF, the psychiatric condition still plays a role, despite the fact that the effect is weakened. These results are similar with the conclusions obtained in Henry and Crawford's^7^ meta-analytic review of VF in schizophrenia.

The absence of significant differences in performance between participants affected by schizophrenia and bipolar disorder, with and without psychotic symptoms, would imply the existence of deficits in executive functions in all these psychiatric conditions.

Although the fact that years of education is a more influential parameter than psychiatric diagnosis for the performance on a PVF test, this variable does not play a main role in the performance of SVF. This difference in the PVF could be due to a cognitive reserve factor,^38^ especially comparing the almost null relations given in control subjects. Nevertheless, the correlations diminish substantially in the case of bipolar psychosis patients, so it is necessary to interpret them carefully. These results emphasize the need to carry out more studies on the effect of education on VF to be able to perform the necessary adaptations when using them as instruments of evaluation, specially the phonemic task.

In general, the data from this study suggest that SVF tests seem to be better evaluation tools, as they are more sensitive to executive dysfunction and less dependent on the education level of the patient than the PVF tests for individuals suffering from schizophrenia and bipolar disorder with and without psychotic symptoms.
